# Green Extraction of Carotenoids from Pumpkin By-Products Using Natural Hydrophobic Deep Eutectic Solvents: Preliminary Insights

**DOI:** 10.3390/molecules30030548

**Published:** 2025-01-25

**Authors:** Lucia Sportiello, Emanuele Marchesi, Roberta Tolve, Fabio Favati

**Affiliations:** Department of Biotechnology, University of Verona, Strada Le Grazie 15, 37134 Verona, Italy; lucia.sportiello@univr.it (L.S.); emanuele.marchesi@univr.it (E.M.); fabio.favati@univr.it (F.F.)

**Keywords:** carotenoid extraction, green chemistry, NaHDESs, pumpkin by-products, sustainable solvents

## Abstract

Natural hydrophobic deep eutectic solvents (NaHDESs), composed of natural components like menthol, fatty acids, and organic acids, are sustainable alternatives to conventional solvents for extracting carotenoids from agro-industrial by-products. This study assessed the performance of nine NaHDESs for extracting β-carotene from pumpkin peels, identifying DL-menthol/lactic acid (1:2) as the most effective solvent, achieving a yield of 0.823 ± 0.019 mg/mL of β-carotene, corresponding to 93.95% of the yield obtained using acetone. Optimization through Box–Behnken design (BBD) and response surface methodology (RSM) established ideal extraction conditions: a molar ratio of HBA:HBD at 1:4, a solvent-to-sample ratio of 26:1, and an extraction time of 30 min. These conditions maximized β-carotene recovery while minimizing energy consumption and process costs. Using NaHDESs facilitates the valorization of food waste, achieving extraction efficiencies of up to 25.05% of the theoretical carotenoid content in pumpkin peels. Their high performance and environmentally friendly profile underscore the potential of NaHDESs as sustainable alternatives to conventional solvents.

## 1. Introduction

Modern food processing industries face the challenge of developing sustainable technologies that maximize resource utilization while minimizing environmental impact. A growing focus has been placed on valorizing the food industry by-products, such as pumpkins, which are rich in bioactive compounds with potential health benefits and diverse industrial applications [1]. Global pumpkin production reach approximately 22.8 million tons in 2022, with China leading as the top producer at 7.3 million tons [2]. While the pulp is widely used, other components such as seeds, peels, and fibrous strands are frequently discarded despite their high nutritional and bioactive potential [3,4]. Pumpkin peels, in particular, are a valuable source of carotenoids, natural antioxidants essential for photosynthesis, and are recognized for their health benefits, including reducing the risk of chronic diseases and supporting immune health. Among various pumpkin varieties, carotenoid content shows considerable variation, with β-carotene ranging from 0.06 to 154.76 mg/100 g, α-carotene from 0 to 10.20 mg/100 g, and lutein from 0 to 17 mg/100 g [5,6,7]. These attributes highlight the potential of carotenoids for applications in the food, pharmaceutical, and cosmetic fields.

Typically, carotenoid extraction is performed with organic solvents such as acetone, ethanol, and hexane. Although effective due to their compatibility with hydrophobic compounds, these solvents have significant disadvantages, including toxicity, environmental hazards, and high energy requirements associated with elevated temperatures and complex purification processes [8,9]. As a result, there is a growing demand for more environmentally friendly and sustainable extraction techniques. Deep eutectic solvents (DESs) have emerged as an innovative approach for extracting valuable bioactive compounds from agro-industrial residues [10,11]. DESs are mixtures of two or more components that form a solvent with a melting point lower than their individual components, offering customizable properties for various applications [10]. They have been successfully used to recover bioactive compounds such as polyphenols, proteins, and carotenoids [12,13], offering advantages due to reduced toxicity, lower energy requirements, and simpler preparation processes compared to traditional methods [14,15]. A commonly recognized challenge in using DES is separating bioactive compounds from the solvent to obtain dry extracts. Several methods have been reported in the literature to facilitate this process, including preparative high-performance liquid chromatography, high-speed counter-current chromatography, solid-phase extraction, column chromatography, pressurized liquid extraction, supercritical fluid extraction, macroporous resin-based techniques, and switchable hydrophilicity solvents [16,17,18,19]. Despite their effectiveness, large-scale industrial use of these techniques is limited by their labor-intensive nature and high costs [19]. However, this limitation can be turned into an advantage, as specific DESs can increase the value of the final product. For example, a carotenoid-enriched DES extract has been successfully integrated into a spreadable chocolate formulation [20]. At the same time, an optimized propolis-rich DES has been employed to produce a cosmetic cream [21]. Hydrophobic natural deep eutectic solvents (NaHDES) based on fatty acids have demonstrated high efficiency in extracting β-carotene [16,22,23,24]. These solvents demonstrated high extraction efficiencies, with yields ranging from 151.41 µg/mL for β-carotene from pumpkin to 653.5 mg/100 g fresh weight for total carotenoid content from orange peels. NaHDESs also exhibited superior antioxidant capacity compared to conventional organic solvents [22,24].

Furthermore, carotenoids extracted using NaHDESs showed improved stability during storage [17,23]. Optimization of extraction parameters, such as solvent-to-solid ratio, extraction time, and temperature, using response surface methodology and ultrasound-assisted extraction, significantly enhanced carotenoid yields [22,24]. These findings highlight the potential of NaHDESs as sustainable and efficient alternatives to conventional solvents for carotenoid extraction. Given the wide variability of solvents that can be obtained by combining different hydrogen bond acceptors (HBAs) and hydrogen bond donors (HBDs), it is valuable to further investigate the potential of other solvents derived from substances with additional properties (e.g., antimicrobial and antioxidant properties).

This study evaluated nine NaHDESs composed of DL-menthol, thymol, camphor, and lactic and decanoic acid for their efficiency in extracting carotenoids from pumpkin peels. After selecting the most effective NaHDES, extraction conditions were optimized using a BBD combined with RSM. This approach aimed to enhance carotenoid recovery by employing advanced statistical optimization and refining the extraction process for greater efficiency and sustainability.

## 2. Results and Discussion

### 2.1. Carotenoid-Rich NaHDES Extracts Preparation

In this study, nine natural and food-grade NaHDESs, previously characterized for their physicochemical properties by Sportiello et al. [20] were prepared using specific molar ratios, as outlined in Table 1. These NaHDESs were composed of monoterpenes (DL-menthol, thymol, camphor) and carboxylic acids (lactic acid and decanoic acid).

**Figure 1:**
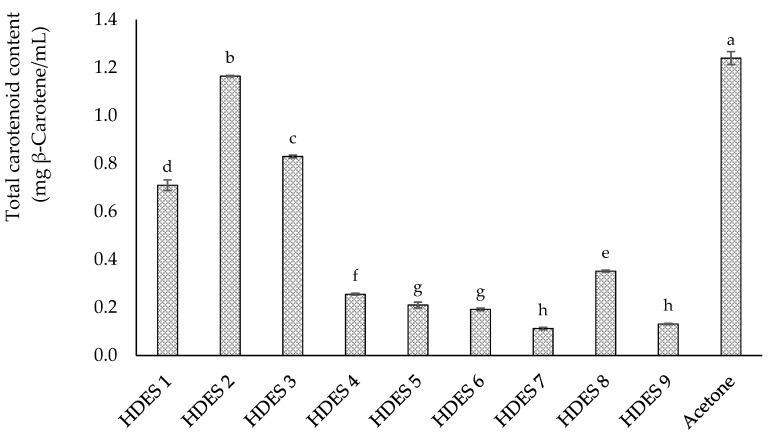
Total carotenoid content extraction obtained using HDES 1 (DL-menthol/lactic acid 1:1), HDES 2 (DL-menthol/lactic acid 1:2), HDES 3 (DL-menthol/lactic acid 8:1), HDES 4 (DL-menthol/decanoic acid 1:1), HDES 5 (DL-menthol/decanoic acid 6.5:3.5), HDES 6 (thymol/DL-menthol 1:1), HDES 7 (thymol/DL-menthol 1:2), HDES 8 (thymol/decanoic acid 3:2), HDES 9 (camphor/decanoic acid 1:2). Data with different superscripts in the same column differ significantly according to Tukey’s test at *p* < 0.05.

The prepared NaHDESs were tested for their efficiency in extracting carotenoids from pumpkin peels. As a reference, an extraction under the same experimental conditions using acetone as the solvent was also carried out. Furthermore, exhaustive extractions were conducted to compare each solvent’s extraction efficiency to the maximum extraction yield obtainable from each matrix. The extraction efficiency of the investigated NaHDESs is shown in Figure 1.

The carotenoid extraction efficiencies from pumpkin peels ranged from 0.710 mg β-carotene/mL to 1.165 mg β-carotene/mL, with HDES 2 demonstrating the highest efficiency among all NaHDES formulations tested. The combination of DL-menthol and lactic acid proved to be the most effective hydrogen bond acceptor (HBA) and hydrogen bond donor (HBD) pair for extracting carotenoids from pumpkin peels, including enhanced polarity modulation, reduced viscosity, and better compatibility with the lipophilic nature of carotenoids. These characteristics likely facilitated the more effective solubilization and stabilization of carotenoids during extraction, leading to their superior performance compared to other NaHDES formulations. In addition, the outcomes of this investigation are consistent with prior research focused on extracting carotenoids from by-products. Specifically, a recent study conducted on carrot by-products has demonstrated how the presence of lactic acid influences the density of NaHDES, improving its effectiveness in carotenoid extraction [20]. Compared with the results reported by Stupar et al. [16] on pumpkin peels, our study achieved values up to six times higher (Caprylic acid:Capric acid 3:1 = 0.2 mg/mL vs. DL-menthol:Lactic acid 1:2 = 1.165 mg/mL). These differences may be attributed to the distinct properties of the DES used, in addition to factors such as the maturity stage and variety of the pumpkin. Despite the highly apolar nature of β-carotene, which typically suggests better solubility in less polar solvents, the DL-menthol:Lactic acid (1:2) DES exhibited superior extraction performance compared to the Caprylic acid:Capric acid (3:1) DES used by Stupar et al. [16]. One plausible explanation lies in the significantly lower viscosity of the DL-menthol:Lactic acid DES (54.821 mPa·s at 20 °C, as reported in our previous study [20]), which likely facilitates better penetration into the plant matrix and more efficient release of β-carotene. By contrast, the viscosity of the Caprylic acid:Capric acid DES remains unreported, but it is reasonable to assume that higher viscosity or less effective matrix disruption could limit its extraction efficiency.

As expected, extraction with acetone yielded the highest recovery of carotenoids, reaching 1.240 mg β-carotene/mL and surpassing all NaHDES formulations tested. This result is consistent with the well-established role of acetone as a solvent for carotenoid extraction, thanks to its excellent solvation properties for these compounds [22,25]. However, while acetone demonstrated superior performance, the primary aim of this study was to evaluate and compare the efficiency of NaHDESs as greener extraction systems. The acetone-based extraction served solely as a standard to contextualize the performance of these sustainable alternatives. According to this, exhaustive extraction was also conducted with acetone and allowed the obtainment of 4.65 mg β-carotene/mL. In this regard, HDES 2 permitted the recovery of 25.05% of the theoretically available carotenoids and around 93.95% of the amount obtained working with acetone under the same conditions. Given its performance, the DL-menthol and lactic acid combination in a 1:2 molar ratio was selected to optimize the extraction process. To maximize carotenoid recovery, the effects of the HBA:HBD molar ratio, solvent-to-sample ratio, and extraction time on total carotenoid content and β-carotene yield were systematically studied using a BBD combined with RSM.

**Figure 2:**
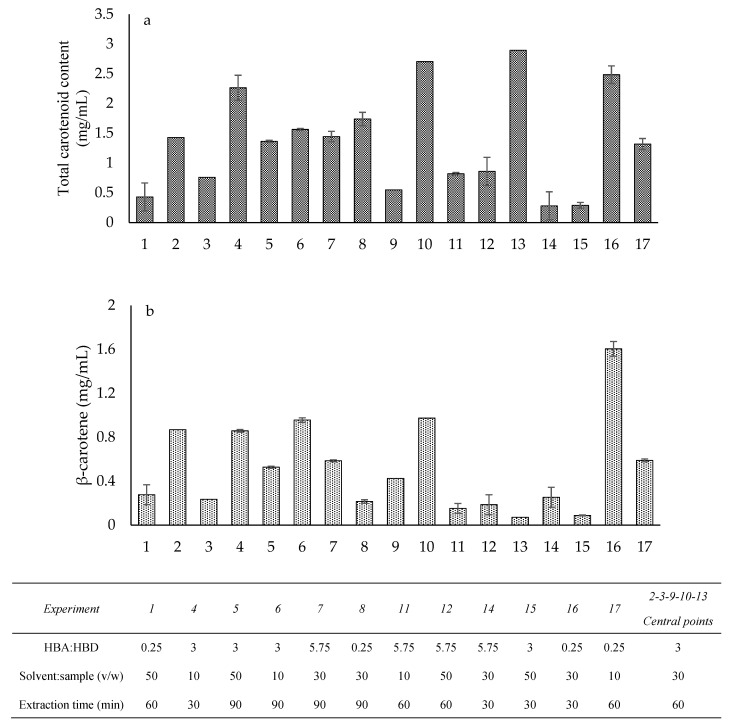
Experimental design responses of the dependent variable total carotenoid content (**a**) and β-carotene (**b**) expressed as mg/mL of extract. The data are presented in histograms showing the mean and standard deviation. The numbers from 1 to 17 are referred to the set of experiments defined by the implemented experimental design.

The results obtained after the extractions proposed by the experimental design were, on average, higher than those observed in the screening phase. Although the matrices used in the experimental phase belonged to the same extract, a possible fluctuation in the carotenoid content can be considered reasonable due to the intrinsic variability. Furthermore, it is worth noting that in this phase, a different extraction temperature was used based on data available in the literature [16,20,26]. Regarding the pigment recoveries, Figure 2a,b reported the amounts of total carotenoids spectrophotometrically assessed and β-carotene yields quantifiable by high-performance liquid chromatography (HPLC) analysis.

Model fitting and statistical verification of the implemented model are reported in Table 2. The obtained data indicated a strong fit of the mathematical models, with R^2^ values exceeding 0.70. Specifically, the models accounted for 88.83% and 96.31% of the variance in total carotenoid content and β-carotene yields from pumpkin peels, respectively. Additionally, the validity of these models in describing the experimental data was supported by the lack of fit *p*-values, which were not significant (*p* > 0.05) across all models tested.

Based on this, the next step involved deriving second-order polynomial regression equations for each dependent variable. This was achieved by incorporating all independent variables and their quadratic interactions while excluding non-significant variables (*p* > 0.05). Considering X_1_ as the HBA:HBD molar ratio, X_2_ as the solvent:sample ratio, and X_3_ as the extraction time, the final equations for total carotenoid content and β-carotene yields were expressed as follows (Equations (1) and (2)):

(1) $$Total\;carotenoid\;content=7.744-5.905\times 10^{-1}\;X_{1}-1.312\times 10^{-1}\;X_{3}+5.227{\times 10^{-2}X}_{1}^{2}+7.042{\times 10^{-4}X}_{3}^{2}+9.833\times 10^{-4}X_{2}X_{3}$$

(2) $$\beta -carotene=3.471\times 10^{-1}-2.043\times 10^{-1}{\;X}_{1}+3.105\times 10^{-2}\;X_{2}+2.051{\times 10^{-2}\;X}_{1}^{2}-2.236\times 10^{-3}{\;X}_{1}X_{2}$$

**Figure 3:**
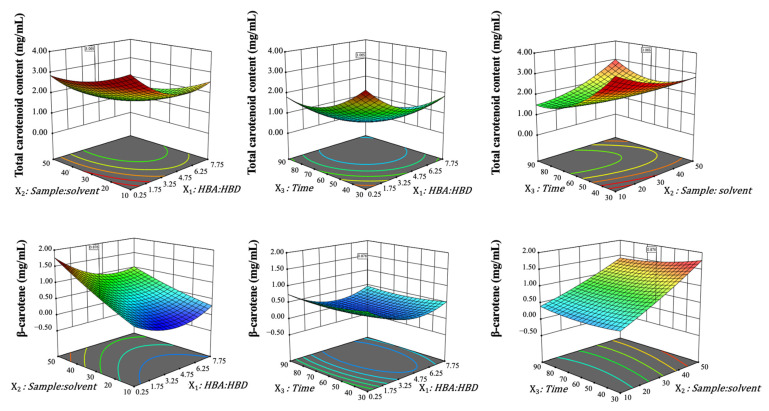
Response surface plots of the fitted polynomial equations (Equations (1) and (2)) for the responses of the extraction from pumpkin peel.

According to the given equations, surface response plots were generated to visualize both the main effects and interactions between two or more independent variables on the responses. These plots, which depict the relationship between two variables at a time while holding the third at its optimal level, are illustrated in Figure 3.

Using HDES 2 (DL-menthol/lactic acid), all surface plots consistently showed that higher yields of β-carotene and total carotenoid content were achieved when the solvent had the lowest HBA:HBD molar ratio (1:4), suggesting that reducing the HBA ratio enhances the solvent’s extraction capacity. These results are consistent with the negative effect of increasing X_1_ (HBA:HBD ratio) on carotenoid yield, as indicated by the linear term in the equation. For extraction time (X_3_) and solvent:sample ratio (X_2_), these variables influenced the results in distinct ways. The plots revealed an increase in total carotenoid yield with short extraction times, as predicted by the quadratic relationship between X_3_ and carotenoid yield. However, this had no significant effect on β-carotene recovery, which is consistent with the weaker influence of X_3_ on β-carotene in the second equation. Lower solvent:sample ratios increased total carotenoid yield, consistent with the positive effect of X_2_ on carotenoid production, while causing a decrease in β-carotene recovery, consistent with the negative interaction between X_1_ and X_2_ on β-carotene yield.

### 2.2. Validation of the Prediction Model

Lastly, utilizing the response data for the total carotenoid content and β-carotene in conjunction with the model parameters determined with BBD, the maximization of all the dependent responses has been carried out using the desirability function, with values ranging from 0 (completely undesirable response) and 1 (fully desirable response). The identified optimal values for the extraction processes were HBA:HBD molar ratio equal to 0.25 (DL-menthol/lactic acid 1:4), solvent-to-sample ratio 26.21 and extraction time 30 min, with the desirability at 0.75, by minimizing both the variables X_2_ and X_3_. As reported above, high solvent:sample ratio values are preferable. However, considering the substantial economic advantages obtainable when a solvent:sample ratio of 10:1 is utilized, especially at the industrial level, the limited decrease in yield was considered an acceptable compromise. The models’ validation was obtained by carrying out the extractions with the identified settings. The fit percentage was found to be very high (>90%) for all the investigated responses, confirming the very good fit of the selected models for analyzing the experimental data (Table 3).

## 3. Materials and Methods

### 3.1. Standards, Reagents and Solvents

Camphor (>96%), DL-menthol (≥98.0%), decanoic acid (≥98.0%), lactic acid (>90%), and thymol (≥98.5%) were used for the NaHDES preparation. For HPLC analysis, acetone (≥99.8%), methanol (≥99.9%), and β-carotene standard (96.4%) were also used. All chemicals were obtained from Merck KGaA (Darmstadt, Germany), and ultrapure water was provided by a Milli-Q system (Millipore, Billerica, MA, USA).

### 3.2. Pumpkin Sample Preparation

Pumpkin peel samples from the processing of fresh pumpkins harvested at their fully mature stage were provided by Ortonuovo Srl (Arbizzano-Santa Maria, Verona, Italy). The samples were washed with tap and deionized water and then dried with absorbent paper. After removing the seeds and comminuting the peels, the samples were freeze-dried using LIO-5P DGT lyophilizer (Vetrotecnica, Padova PD, Italy) and ground with a Polymix PX-MFC 90D mill (Vetrotecnica, Padova PD, Italy). The obtained powder was vacuum-sealed and stored at −20 °C.

### 3.3. NaHDESs Preparation

Natural hydrophobic deep eutectic solvents (NaHDESs) consist of a mix of hydrogen bond acceptors (HBA) and hydrogen bond donors (HBD) in different molar ratios. The NaHDESs were prepared following the method outlined by Dai et al. [27], with slight modifications. The HBA and HBD were mixed in a specific molar ratio, as outlined in Table 1, and stirred at 750 rpm under mild heating at 60 °C until a clear, transparent liquid was obtained. The resulting solvents were then cooled to room temperature, and the thermal stability was monitored during storage. These solvents were characterized in a previous study by Sportiello et al. [13].

### 3.4. Extraction of Carotenoids Using NaHDESs

To identify the most efficient NaHDES in the extraction screening tests, 100 mg of lyophilized pumpkin peel powder was mixed with 5 mL of each prepared NaHDES, using a sample-to-solvent ratio of 1:50 (*w*/*v*). The mixtures were vortexed for 60 s, followed by 30 min of continuous mixing at room temperature on a disc rotator (UniLOPMIX2, LLG-Labware, Meckenheim, Germany). Then, the mixture was sonicated for 60 min at 45 kHz (2200 MH S3, SOLTEC, Milan, Italy) and centrifuged at 3900 RCF for 10 min. All procedures were conducted in triplicate and under controlled conditions at 25 ± 2 °C, with samples protected from light to minimize carotenoid photodegradation. To benchmark the extraction efficiency of NaHDESs against a conventional organic solvent, a parallel extraction was performed using acetone under identical conditions. Additionally, exhaustive extraction was performed to evaluate the efficiency of each solvent compared to the maximum theoretical yields obtained with acetone.

### 3.5. Extraction Efficiency of Total Carotenoid Content and β-Carotene

The extraction efficiency of total carotenoid content from pumpkin peels was monitored using UV–Vis spectrophotometry, while HPLC-DAD analysis quantified the β-carotene yields. The total carotenoid content in the NaHDESs extracts was determined at 450 nm, following the Beer–Lambert law, as per the method outlined by Ordonez-Santos et al. [28]. A V-750 UV–Visible Spectrophotometer (Jasco, Portland, OR, USA) was employed, with samples diluted 1:5 in acetone (*v*/*v*) for the analysis; the corresponding solvent served as blanks for each extract quantification. Carotenoid concentration was determined considering the extinction coefficient for β-carotene in acetone (2500) and expressed in mg of β-carotene per mL of extract.

### 3.6. HPLC-DAD Analysis

The β-carotene content in the pumpkin peel extract was analyzed by HPLC using an LC-4000 HPLC system with a PDA detector (MD-4010, Jasco, Portland, OR, USA) and a C30 column (4.6 × 250 mm, 5 μm, YMC Inc., Wilmington, NC, USA). Peak separation was achieved using a solvent gradient, with solvent A (H_2_O/MeOH 20/80, *v*/*v*) and solvent B (acetone/MeOH 1:1, *v*/*v*) at a flow rate of 1 mL/min. The gradient profile was as follows: 25% B from 0 to 4 min, 100% B from 4 to 10 min, 100% B from 10 to 25 min, and 25% B from 25 to 36 min, based on a slightly modified method by Šeregelj et al. [29]. Analytical samples were diluted in acetone (1:2, *v*/*v*) and filtered through a 0.45 µm PTFE filter (Frisenette, Knebel, Denmark). β-carotene was identified by comparing the retention times and absorption spectra with an external standard. Pigments (mg/mL of extract) were quantified using the external standard technique and specific calibration curves.

### 3.7. Extraction Optimization

The most effective NaHDES for extracting carotenoids from pumpkin peels was chosen after the preliminary screening. A three-factor, three-level BBD combined with RSM was employed to enhance the extraction process further and maximize efficiency. In this optimization, three independent variables were examined: the molar ratio of hydrogen bond acceptor to hydrogen bond donor (HBA) (X_1_), the solvent-to-sample ratio (X_2_), and the extraction time (X_3_). These factors were studied at three levels, coded as −1, 0, and +1. Following the method outlined by Purohit and Gogate [26] and Stupar et al. [16], the extraction temperature was held constant at 50 °C. The experimental ranges and levels for these variables in BBD are summarized in Table 4.

In Table 5, the experimental design with the combinations of the independent variables studied at three levels is reported. The total carotenoid content, spectrophotometrically calculated, and the yields of β-carotene, obtained by HPLC analysis, were selected as dependent response variables to optimize the extraction process. A second-order model was adopted to explain the relationship between the dependent responses and the factors. The extraction tests were conducted in triplicate to ensure model strength except for the central points.

### 3.8. Statistical Analysis

The statistical analysis of the data obtained during the screening phase was conducted using XLSTAT Premium software (Version 2020.3.1, Addinsoft, Paris, France) with one-way ANOVA. Significant differences between means were identified through Tukey’s HSD (Honestly Significantly Different) test at a 95% confidence level. For process optimization, the dependent variables were analyzed using BBD. The final models included terms with a significance level of less than 0.05 (*p* < 0.05) and terms necessary to maintain the model’s hierarchical structure. Multiple linear regression analysis was used to develop the best-fitting model, with its adequacy evaluated based on the *p*-value, lack of fit, coefficient of determination (R^2^), and adjusted R^2^ (R^2^adj). Optimal extraction conditions were determined using the desirability function to maximize the efficiency of all target responses while applying constraints to X_2_ (solvent-to-sample ratio) and X_3_ (extraction time) to minimize process costs. Model adequacy was verified by performing extractions under the predicted optimal conditions in triplicate. The experimental design, optimization process, and creation of three-dimensional response surface plots were carried out using Design Expert software (Version 8.0.7.1, Stat-Ease Inc., Arden Hills, MN, USA).

## 4. Conclusions

This study evaluated the efficiency of NaHDESs for carotenoid extraction from pumpkin peels, a by-product of pumpkin processing. Nine different NaHDESs were selected and characterized to determine their suitability for carotenoid extraction. These solvents were screened for their ability to extract β-carotene. The screening process identified the most effective NaHDES obtained from a mixture of DL-menthol and lactic acid in a molar ratio of 1:2. The extraction process using this solvent was subsequently optimized to maximize extraction efficiency. The optimization was achieved using BBD and RSM, robust statistical tools that allow tuning multiple variables to achieve optimal yield. Under the optimized conditions, the NaHDES system achieved an extraction yield of 0.823 ± 0.019 mg/mL of β-carotene, demonstrating performances that match traditional acetone-based methods. In addition, NaHDESs offer several additional advantages from a green chemistry perspective, including reduced energy requirement due to their low volatility and milder operating conditions and lower environmental impact due to the biodegradability and non-toxicity of the components used. These characteristics position NaHDESs as a promising sustainable alternative to conventional solvents, especially for industries prioritizing environmentally friendly practices in extracting natural compounds from food wastes, agricultural by-products, and other biomass sources. Overall, this study increases the knowledge of NaHDESs and their use for carotenoid extraction. It highlights their potential use in food and pharmaceutical industries to extract bioactive compounds from by-products. The sustainability and efficiency of NaHDESs are in line with the growing demand for environmentally responsible extraction methods, further solidifying their role as a transformative solution in these sectors.

## Figures and Tables

**Table 1:** HBAs/HBDs, molar ratios of NaHDESs, and carotenoid content of the obtained extracts (mg/mL) as determined by UV–Vis spectrophotometry after extraction during the screening step.

NaHDESs	HBA/HBD	Molar Ratio
HDES 1 HDES 2 HDES 3	DL-menthol/lactic acid	1:1 1:2 8:1
HDES 4 HDES 5	DL-menthol/decanoic acid	1:1 6.5:3.5
HDES 6 HDES 7	Thymol/DL-menthol	1:1 1:2
HDES 8	Thymol/decanoic acid	3:2
HDES 9	Camphor/decanoic acid	1:2

**Table 2:** Coded second-order coefficients, determination coefficients (R^2^ and R^2^_adj_), lack of fit and *p* values of the fitted models on the investigated responses.

		Total Carotenoid Content (mg/mL)	β-Carotene (mg/mL)
**Constant**	$\beta_{0}$	7.744 ***	3.471 × 10^−1^ ***
**Linear**	$\beta_{1}$	−5.905 × 10^−1^ *	−2.043 × 10^−1^ **
	$\beta_{2}$	−9.674 × 10^−2^	3.105 × 10^−2^ ***
	$\beta_{3}$	−1.312 × 10^−1^ *	−9.719 × 10^−3^
**Quadratic**	$\beta_{11}$	5.227 × 10^−2^ **	2.651 × 10^−2^ ***
	$\beta_{22}$	7.688 × 10^−4^	1.557 × 10^−4^
	$\beta_{33}$	7.042 × 10^−4^ *	9.842 × 10^−5^
**Interaction**	$\beta_{12}$	−4.500 × 10^−4^	−2.236 × 10^−3^ *
	$\beta_{13}$	8.000 × 10^−4^	1.307 × 10^−4^
	$\beta_{23}$	9.833 × 10^−4^ *	−1.599 × 10^−4^
**R^2^**		0.8883	0.9631
**R^2^_adj_**		0.7447	0.9158
**Lack of Fit**		0.0564	0.1711
***p*** **-value**		0.0126	0.0003

*, **, *** significantly different at *p* < 0.05, *p* < 0.01, and *p* < 0.001, respectively. β_0_: constant; β_1_: coefficient of the linear effect of HBA:HBD molar ratio; β_2_: coefficient of the linear effect of solvent:sample ratio; β_3_: coefficient of the linear effect of extraction time; β_11_: coefficient of the quadratic effect of HBA:HBD molar ratio; β_22_: coefficient of the quadratic effect of solvent:sample ratio; β_33_: coefficient of the quadratic effect of extraction time; β_12_: interaction coefficient of HBA:HBD molar ratio and solvent:sample ratio; β_13_: interaction coefficient of HBD:HBA molar ratio and extraction time; β_23_: interaction coefficient of solvent:sample ratio and extraction time.

**Table 3:** Predicted and actual experimental values of the investigated responses under the optimal extraction conditions.

	Predicted Value (mg/mL)	Experimental Value (mg/mL)	% Fit
Total carotenoid content	3.065	2.995 ± 0.021	97.716%
β-carotene	0.876	0.823 ± 0.019	93.950%

**Table 4:** Experimental ranges with the coded and natural values of the independent variables for carotenoid extraction from pumpkin peels (HDES 2).

Independent Variables	Levels		
	−1	0	+1
(X_1_) HBA: HBD (DL-menthol/lactic acid) molar ratio	0.25	4	7.75
(X_2_) Solvent:sample ratio (*v*/*w*)	10	30	50
(X_3_) Extraction time (min)	30	60	90

**Table 5:** Experimental design with independent variables.

Experiment No.	HBA:HBD Molar Ratio	Solvent:Sample Ratio (*v*/*w*)	Extraction Time (min)
1	0.25	50	60
2	3	30	60
3	3	30	60
4	3	10	30
5	3	50	90
6	3	10	90
7	5.75	30	90
8	0.25	30	90
9	3	30	60
10	3	30	60
11	5.75	10	60
12	5.75	50	60
13	3	30	60
14	5.75	30	30
15	3	50	30
16	0.25	30	30
17	0.25	10	60

## Data Availability

The original contributions presented in this study are included in the article.
